# Completion of multiple-dose travel vaccine series and the availability of pharmacist immunizers: A retrospective analysis of administrative data in Alberta, Canada

**DOI:** 10.1371/journal.pone.0211006

**Published:** 2019-01-23

**Authors:** Sherilyn K. D. Houle, Dean T. Eurich

**Affiliations:** 1 School of Pharmacy, University of Waterloo, Waterloo, Ontario, Canada; 2 School of Public Health, University of Alberta, Edmonton, Alberta, Canada; University of Campania, ITALY

## Abstract

Pharmacists in a number of countries are being trained in the administration of injections with the aim of improving access and adherence to vaccinations. However, little is known about population-level adherence to multiple-dose travel vaccines, and whether the availability of pharmacist immunizers is associated with adherence. Health administrative data from Alberta, Canada, from April 2008 to May 2017 identified adults dispensed at least one vaccine for hepatitis A, hepatitis B, Japanese encephalitis, or rabies. Individuals were coded as completers or non-completers of the vaccine series based on the number of doses dispensed over a time period comprising the duration of the standard series plus 6 months to account for late doses. The association between the proportion of Alberta pharmacists with injection authorization (according to pharmacist registration data) and completion of vaccine series was assessed using linear regression. Over the study period, 24,164 patients initiated a vaccine series for hepatitis A monovalent, 195,480 for hepatitis B monovalent, 169,802 for combined hepatitis A&B, 1,726 for Japanese encephalitis, and 1,908 for rabies. There were fewer than 5 individuals receiving Japanese encephalitis vaccine per year from 2008–2010 or rabies vaccine from 2008–2009. While statistically significant positive associations were seen across all vaccines except for Japanese encephalitis, the magnitude of these associations was small. Each 1% increase in the proportion of injections-authorized pharmacists saw a corresponding increase in the proportion of individuals with completed vaccine series by 0.31% for hepatitis A monovalent, 0.19% for hepatitis B monovalent, 0.22% for combined hepatitis A&B, and 0.21% for rabies. This may suggest that challenges remain with implementing reminder systems to ensure adherence among travellers. Strategies to develop or improve patient and clinician reminder systems in pharmacies for travel vaccines should therefore be explored.

## Introduction

Healthcare providers, including pharmacists, are trusted sources of information on immunizations [1–3]; indeed, recommendation from a healthcare professional to be vaccinated is strongly associated with a patient’s decision to be vaccinated [4–5]. Greater availability of evidence-based information from a health professional and convenient access to vaccination services may help address concerns related to vaccine hesitancy and suboptimal vaccination rates [6]. Due to their geographic accessibility, extended hours, and availability often without an appointment [7], pharmacists are widely consulted healthcare professionals. In an effort to improve patient access to immunizations by leveraging this accessibility and convenience, the state of Washington became the first jurisdiction to train pharmacists to administer vaccines in 1994. Currently, pharmacists in all US states and a number of other countries (Argentina, Australia, Canada, Philippines, South Africa, and the United Kingdom, among others) authorize some level of vaccination in pharmacies and/or by pharmacists [8]. Legislation on specific vaccines that can be administered and eligible patient populations highly varies by region, but appears to be expanding from initial restrictions to influenza vaccinations for adults towards broader vaccine product eligibility and even adolescent and pediatric vaccination [9–10].

A study of over six million vaccinations administered in community pharmacies in the United States found that one-quarter were provided on evenings, weekends, or holidays, when more traditional vaccine providers are often unavailable [11]. Patients receiving vaccinations from pharmacists have also reported high satisfaction with this service [12]. A recent systematic review and meta-analysis found that vaccination rates improved when a pharmacist was involved in the immunization compared to traditional non-pharmacist providers. Pooled analyses of two randomized controlled trials found that patients randomized to pharmacist administration were more than two times likely to receive vaccination (relative risk, RR, 2.64, 95% confidence interval, CI, 1.81, 3.86) than those randomized to traditional providers [13]. However, all studies included in this review evaluated only outcomes related to influenza and pneumococcal vaccines. No published work to date has evaluated the impact of pharmacists-as-immunizers on travel vaccine uptake.

Efforts to mitigate exposure to, and the risk of developing, vaccine-preventable diseases is of great importance for travellers [14]. A surveillance study of 10 years of data among over 37,000 ill travellers found that 508 presented with a vaccine-preventable disease [15]; however, it is recognized that this may represent an under-report as data was collected from select clinics only, and may not have captured individuals with mild or self-limited illnesses or who received care from a non-participating clinic or hospital. Even if overall incidence rates are low [16], the consequences of these illnesses can be significant. In addition to morbidity and mortality among affected individuals, imported disease has been found to impede disease eradication in susceptible countries (e.g., polio) and to contribute to outbreaks in the travellers’ home country upon return (e.g., influenza, measles, meningitis) [14]—each of which have both humanistic and economic impacts. Many travel clinics administer vaccines at the time of a pre-travel consultation. However, other healthcare providers such as family physicians may not carry stock of travel vaccines if they do not perform pre-travel consultations often. In these cases, patients are generally issued prescriptions, which are then dispensed by a community pharmacy. If the vaccine could not be administered at the pharmacy, these products would be brought back to the physician’s office by the patient for injection, with the potential for cold chain breaches during storage and transport by the patient. However, the greatest potential value of community pharmacy-provided vaccinations may be realized with multiple-dose vaccine regimens, as patients can benefit from the convenient operating hours of pharmacies for subsequent doses or boosters.

Adherence with multiple-dose vaccine regimens among travellers is suboptimal. Among 3576 travelers to hepatitis A endemic countries in Africa, Asia, and South and Central America and who reported receiving vaccination against hepatitis, 37% reported being adherent to a 2-dose monovalent hepatitis A regimen, and 27% were adherent to the 3-dose combined hepatitis A&B regimen [14]. Reasons stated for non-adherence included lack of information about vaccine schedules, lack of time, and a need for reminders. Of concern, 61% of those who were only partially vaccinated against hepatitis A, and 84% of those partially vaccinated with combined hepatitis A&B felt they were fully vaccinated. Similar issues with incomplete vaccine series among adults have been observed among the general population for hepatitis A [15], hepatitis B [15], varicella [15], tickborne encephalitis [16], and tetanus [17].

In Alberta, Canada, legislation was enacted granting pharmacists the authorization to administer vaccines and drugs by injection in 2008, following the successful completion of a training program on immunization and current certification in First Aid and cardiopulmonary resuscitation. There are no restrictions on vaccine type or product that can be administered; however, pharmacists cannot immunize patients under age 5 (or age 9 for influenza vaccine, specifically) [18–19].

The objectives of this study are to examine adherence to multiple-dose travel vaccine schedules in Alberta, and to determine if an association exists between the proportion of licensed pharmacists authorized to immunize and the proportion of patients completing the vaccine series.

## Methods

### Study design

Retrospective observational study using the Alberta Health administrative databases.

### Data source

Alberta Health administrative datasets, including the Population Registry and Pharmaceutical Information Network (PIN) [20], from April 1, 2008 to March 31, 2017. Dispensing data from the PIN was linked to each individual’s demographic data within the Population Registry using their Personal Health Number as their unique identifier. As of April 2017, approximately 95% of Alberta pharmacies submit dispensing data to the PIN [20]. Information on the number of pharmacists with authorization to administer drugs or vaccines by injection was obtained from publicly-available reports on the Alberta College of Pharmacy website [21].

### Inclusion and exclusion criteria

All Alberta residents age 18 years or older, who were dispensed at least one eligible vaccine product as an incident prescription within the study period, were included. Eligible products included those that provided protection against hepatitis A (monovalent), hepatitis B (monovalent), combined hepatitis A&B, Japanese encephalitis, or rabies. These products were selected as they are commonly indicated for protection against the disease during travel, and because their primary series requires more than 1 vaccine dose to provide protection. Vaccines usually received in childhood but requiring boosters in adulthood (e.g., tetanus), or requiring annual doses (e.g., influenza) were excluded. Eligible vaccines were identified using the drug identification number (DIN) for products available in the Canadian market [22]. In total, 14 unique vaccine products were eligible, consisting of 4 for protection against hepatitis A, 5 for hepatitis B, 2 for combined hepatitis A&B, 1 for Japanese encephalitis, and 2 for rabies. Subject groups were created separately for each DIN; therefore, the same individual may be a member of more than one group if they received more than one of the eligible vaccines. Incident prescriptions were ensured by applying a 6-month washout period prior to the first recorded dispense per person, per product.

### Outcome measures

The primary outcome was the proportion of individuals dispensed a sufficient number of vaccine doses to complete the recommended series for each vaccine-preventable disease. The secondary outcome was the association, if any, between the proportion of individuals completing the vaccine series and the proportion of pharmacists authorized to administer injections. To account for late doses, and because administrative data cannot tell us if a usual or accelerated regimen was intended, the follow-up period applied to each vaccine was the longest recommended dosing schedule according to Canadian product monographs plus an additional 6 months, and a regimen was considered to be completed if the minimum number of doses required has been received within that period. For example, combined hepatitis A&B vaccine has a usual schedule of 0, 1, and 6 months, or an accelerated schedule of 0, 7 days, and 21 days with a 12-month booster [23]. The longest regimen (12 months for accelerated schedule) plus 6 months would mean that the follow-up period for subjects in this vaccine group will be 18 months. As the usual schedule requires 3 doses and the accelerated schedule requires 4 doses, the receipt of at least 3 doses within 18 months was considered to be a complete series. Follow-up periods and minimum number of required doses applied per vaccine are described in Table 1.

**Table 1:** Follow-up periods and required doses applied per vaccine for series completion.

Vaccine	Follow-Up	Number of Doses
Hepatitis A (monovalent)	18 months	2
Hepatitis B (monovalent, Engerix-B)	18 months	≥3
Hepatitis B (monovalent, Recombivax HB)	12 months	3
Combined hepatitis A&B	18 months	≥3
Rabies	7 months	3
Japanese encephalitis	7 months	2

When multiple vials or doses of a vaccine were dispensed by a pharmacy on the same date to the same patient, it was assumed that each of those doses was administered according to the vaccine schedule. For scenarios where different brands of vaccine for the same indication were used to complete a vaccine series in the same patient, guidance on the interchangeability of products from the CDC Yellow Book and the Committee to Advise on Tropical Medicine and Travel (CATMAT) [24–25] were consulted. When interchangeability was appropriate, individuals were counted as receiving all doses across each brand of vaccine for that disease. Furthermore, individuals receiving a monovalent hepatitis A or hepatitis B dose followed by the remainder of doses required for series completion using the combined hepatitis A&B vaccine (brand name Twinrix) were coded as completing the series for the monovalent regimen.

### Statistical analyses

For each year between 2008 to 2016, the proportion of patients completing and not completing each travel vaccine series was computed. For this, the denominator was determined as the total number of patients who received at least one dispensation for the travel vaccine regimen and the numerator was defined as the total number of patients completing or not completing the series within the allotted time period as defined above. Linear regression was then used to evaluate the association between the total number of pharmacists eligible to administer travel vaccines and the proportion of patients completing each series year over year. For this analysis, the total number of pharmacists eligible per year was used as the independent variable while the proportion of patients completing each series per year was used as the dependent variable. Separate analyses were conducted for each specific vaccine regimen.

### Ethics

Research ethics approvals were obtained from the Office of Research Ethics at the University of Waterloo, and the Health Research Ethics Board at the University of Alberta. As all data were anonymized by Alberta Health (the data custodian) through the use of a scrambled recipient unique lifetime identifier as the only identifier, informed consent from each individual was not required for ethics approval from either institution.

## Results

**Fig 1:**
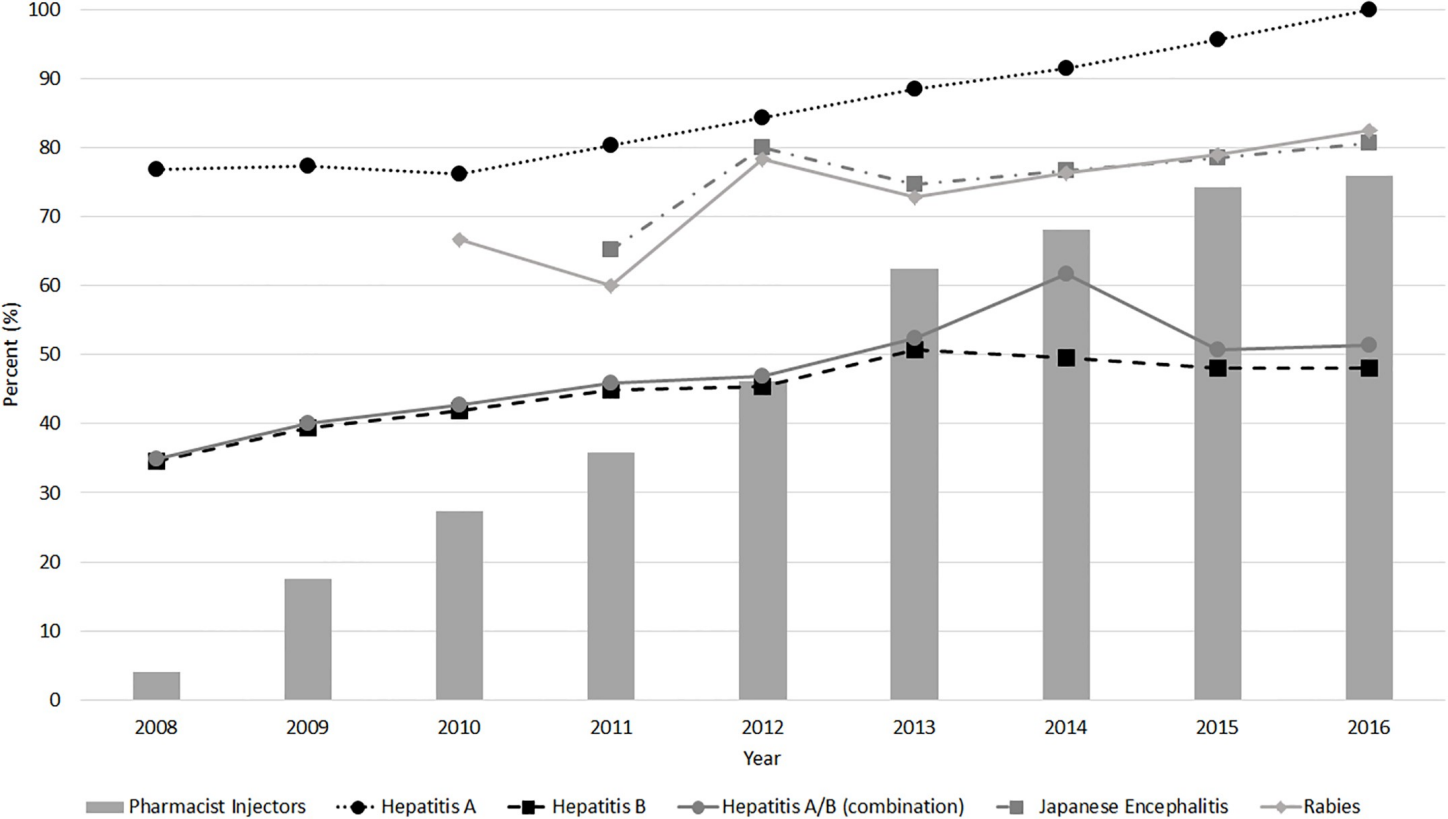
Percentage of pharmacists authorized to administer injections and percentage of series completion per vaccine, per year.

Over the study period, 24,164 patients initiated a vaccine series for hepatitis A monovalent, 195,480 for hepatitis B monovalent, 169,802 for combined hepatitis A&B, 1,726 for Japanese encephalitis, and 1,908 for rabies. Data on series completes and incompletes based on year of incident prescription is summarized in Table 2. As there were fewer than 5 individuals receiving the Japanese encephalitis vaccine per year from 2008–2010 and the rabies vaccine from 2008–2009, data for these cells is not provided. The proportion of Alberta pharmacists with authorization to administer injections and the proportions of individuals initiating each vaccine series who completed it, per year, are illustrated in Fig 1.

**Table 2:** Completion status of vaccine series based on year of incident prescription, and number and proportion of pharmacists authorized to administer injections per year.

	2008	2009	2010	2011	2012	2013	2014	2015	2016
**Vaccine Series Completion**									
**Hepatitis A (monovalent)**									
% complete	76.8%	77.3%	76.2%	80.2%	84.2%	88.5%	91.4%	95.6%	100%
n complete	1359	2570	2157	2187	2496	2785	3165	2794	18
n total	1770	3323	2830	2726	2963	3147	1464	2923	18
**Hepatitis B (monovalent)**									
% complete	34.5%	39.4%	42.0%	44.9%	45.4%	50.7%	49.5%	48.0%	48.0%
n complete	5416	10348	8791	8901	10622	12922	14024	12568	4499
n total	15698	26293	20955	19837	23375	25475	28318	26163	9366
**Combined hepatitis A&B**									
% complete	34.9%	40.0%	42.7%	45.9%	46.9%	52.4%	51.6%	50.7%	51.4%
n complete	5121	9711	8237	8023	9582	11393	12240	10497	3818
n total	14673	24302	19281	17489	20454	21734	23717	20719	7433
**Japanese encephalitis**									
% complete	---	---	---	65.1%	80.0%	74.6%	76.7%	78.4%	80.7%
n complete	---	---	---	28	68	82	204	352	624
n total	---	---	---	43	85	110	266	449	773
**Rabies**									
% complete	---	---	66.7%	60.0%	78.3%	72.9%	76.3%	78.9%	82.5%
n complete	---	---	26	30	47	97	257	396	637
n total	---	---	39	50	60	133	337	502	772
**Pharmacists with Authorization to Administer Injections**									
n authorized	157	711	1137	1535	2044	2842	3239	3755	4069
n total licensed	3885	4058	4152	4277	4431	4550	4759	5055	5363
% authorized	4.0%	17.5%	27.4%	35.9%	46.1%	62.5%	68.1%	74.3%	75.9%

Statistically significant positive associations were seen between the proportion of individuals completing a vaccine series and the proportion of pharmacists authorized to administer injections for each vaccine with the exception of Japanese encephalitis; however, the magnitude of these associations was small (Table 3). Each 1% increase in the proportion of pharmacists authorized to administer injections resulted in increases in the proportion of individuals with completed vaccine series by 0.31% for hepatitis A monovalent, 0.19% for hepatitis B monovalent, 0.22% for combined hepatitis A&B, and 0.21% for rabies. Stated alternatively, this would equate to 2–3 additional patients completing a series per 1000 patients initiating a vaccination series or, in the case of all hepatitis A, an additional 75 patients would have completed among the 24,164 patients who initiated. The clinical importance of these findings is unclear.

**Table 3:** Regression results for association between completion of vaccine series and proportion of registered pharmacists with authorization to administer injections.

Vaccine	Coefficient	Standard Error	R-Squared	p-value
Hepatitis A (monovalent)	0.309	0.040	0.893	<0.001
Hepatitis B (monovalent)	0.188	0.027	0.871	<0.001
Combined hepatitis A&B	0.224	0.023	0.931	<0.001
Japanese encephalitis	0.245	0.129	0.473	0.131
Rabies	0.211	0.066	0.591	0.016

## Discussion

The availability of immunization-authorized pharmacists in Alberta was associated with small, statistically significant increases in the completion rates of vaccine series for hepatitis A and B monovalent vaccines, combined hepatitis A&B vaccine, and rabies vaccine, but not for Japanese encephalitis vaccine. The clinical significance of these findings is unclear, as long-term monitoring for the incidence of these vaccine-preventable diseases was not performed.

A large population-based retrospective study using administrative data for 8.8 million Medical Care Organization enrollees in the United States reported on series completion rates for varicella, hepatitis A, and hepatitis B vaccines within one year of an initial dose. This study observed completion rates in adults of 49–56% for varicella, 25–44% for hepatitis A, and 41–62% for hepatitis B [15]. In another study, prescription dispensing data was used to estimate compliance with the 3-dose vaccination series against tick encephalitis in Germany. Among a sample of 6,796 adults who received an initial dose, second doses were received by 43–54% of individuals (varying depending on age strata), and third doses by 19–27% [26]. The series completion rates observed in our analysis were similar to these previous reports for hepatitis B monovalent vaccine (ranging from 34.5% in 2008 to 50.7% in 2013) and combined hepatitis A&B vaccine (ranging from 34.9% in 2008 to 52.4% in 2013), but were higher for all other vaccines (ranging from 60% for rabies vaccine in 2008 to 100% for hepatitis A monovalent vaccine in 2016).

Previous research identified a significant association between pharmacist density and adult influenza immunization receipt in the United States [27]. In this study, an increase of 1 pharmacist per 1000 individuals in the population was associated with 13% higher odds of an individual receiving the vaccine (95%CI 11–15%) [27]. Our analysis did not account for changes in pharmacist density. Over the study period, the population of Alberta grew by 9% from 33.2 million to 36.2 million [28], while the number of licensed pharmacists with injection authorization—as a new service introduced in 2008—grew exponentially from 157 to 4069 [21]. However, the distribution of these pharmacists across the province was unknown, so we cannot assess whether an association exists between pharmacist distribution and population distribution. Additionally, it must be considered that influenza vaccine differs from other vaccines across a number of aspects. For example, the results of a study suggest that pharmacists may feel less comfortable administering travel vaccines compared to more routine ones such as influenza or pneumococcal vaccines. Prior to passing legislation in Ontario, Canada which expanded pharmacists’ authority to include injecting a number of travel vaccines, some pharmacists expressed discomfort with potentially administering some of the more specialized vaccines such as yellow fever, Japanese encephalitis, typhoid, and rabies [29]. These pharmacists felt their knowledge in travel medicine was too limited to confirm the appropriateness of these vaccines for travellers before administering them, or advise patients on monitoring for adverse reactions to them. This was despite the fact that this legislation simply authorized the administration of these vaccines, which still had to be prescribed by a physician or other authorized prescriber.

Pharmacies have also historically been more involved in administering vaccines with less frequent dosing schedules and/or fewer vaccinations to complete a series (e.g., influenza, herpes zoster, and pneumococcal) than is seen with many travel vaccines, allowing them to be more easily timed to coincide with visits for prescription refills or other services and requiring fewer reminders overall than vaccines indicated for travel-related purposes. To address the added dosing complexity of a number of travel vaccines, reminder strategies at both the pharmacy and patient levels may need to be implemented [30], and may take the form of electronic (e.g., reminder settings within dispensary software or patient apps), written, or direct contact approaches, which have been studied using lay health workers [31] or health professionals [32]. As administrative data was used in our study, we are unable to ascertain which, if any, reminder strategies were used by pharmacies.

A strength of our study is the complete capture of vaccines dispensed by pharmacies in the province, irrespective of whether the patient paid for the vaccine out of pocket or not. However, our study is not without limitations which must be considered when interpreting the results of our study. First, the administrative databases utilized do not require the imputation of the indication for therapy when a product is dispensed. As such, we cannot be certain that each vaccine was ordered specifically for the prevention of illness acquired during travel. Additionally, the directions for use or prescribed regimen were not captured; therefore, we were unable to identify if a regimen for hepatitis B monovalent or combined hepatitis A&B vaccine intended to follow the regular or accelerated dosing schedule. For the purpose of the study, both options were accommodated by considering a patient to have completed the series if they received the minimum number of doses to complete either regimen, and by assigning the longest series as the reference time period for receiving all doses. This lack of directions for use also may result in cases being mis-coded as incomplete if only a single dose was intended for either short-term protection (for example, hepatitis A can provide ≥95% seroprotection after a single dose [33], with subsequent doses intended to provide long-term immunity) or a booster dose when vaccination records were incomplete or titres showed suboptimal protection from previous vaccination. However, this would be expected to be a relatively consistent occurrence year over year, and therefore have negligible impact on our findings.

We were also unable to determine if the vaccines dispensed were subsequently administered by a pharmacist. It is possible that patients could be dispensed a vaccine for injection at a medical or public health clinic, or that vaccine wastage occurred if a patient purchased a vaccine for offsite administration but ultimately did not receive the injection. Furthermore, we cannot be certain that the first dose dispensed by a community pharmacy represents the first dose for a given patient’s vaccine series, or that this data captures all vaccines for travel-related purposes received by Albertans. Travel clinics and medical practices may choose to stock their own supply of vaccines for purchase by patients, which may not be entered into the administrative datasets used in this study. It is possible that the initial dose of a vaccine could be administered at the time of the travel consultation, with subsequent doses received from a community pharmacy. This may be especially likely if the patient had to travel to another community to access the pre-travel consultation service. Finally, the licensure of pharmacists as having authorization to administer injections does not ensure their participation in vaccination programs, or travel vaccinations specifically. Some of these pharmacists may practice in hospitals or primary care clinics where injections are largely administered by nurses, or may practice in community pharmacies that choose not to administer these vaccines but instead focus solely on annual influenza campaigns. Our analysis was unable to exclude pharmacists with injection authorization who did not practice in a community pharmacy or did not regularly administer injections. The inclusion of these non-vaccinating pharmacists in our analyses may therefore underestimate the potential impact of pharmacists who do administer the vaccines included in this study related to adherence.

In summary, while the availability of pharmacist immunizers appears to have had a positive impact on vaccine series completion, the magnitude of this impact has been small, and may signal challenges faced with introducing this service and subsequent reminder systems into community pharmacy practice. Pharmacists have become widely-accessed providers for influenza vaccination, suggesting patient acceptance of and satisfaction with pharmacists as immunization providers. Future research should address patient and pharmacist barriers unique to travel vaccines and other multiple-dose vaccines, including strategies to develop or improve patient and clinician reminder systems in pharmacies.
